# Towards Clinical Translation of LED-Based Photoacoustic Imaging: A Review

**DOI:** 10.3390/s20092484

**Published:** 2020-04-27

**Authors:** Yunhao Zhu, Ting Feng, Qian Cheng, Xueding Wang, Sidan Du, Naoto Sato, Jie Yuan, Mithun Kuniyil Ajith Singh

**Affiliations:** 1 Department of Electronic Science and Engineering, Nanjing University, Nanjing 210023, China; yunhaoz@umich.edu (Y.Z.); coff128@nju.edu.cn (S.D.); 2 Department of Biomedical Engineering, University of Michigan, Ann Arbor, MI 48109, USA; xdwang@umich.edu; 3 Department of Electronic and Optical Engineering, Nanjing University of Science and Technology, Nanjing 210094, China; fengting@njust.edu.cn; 4 Institution of Acoustics, Tongji University, Shanghai 200092, China; Q.cheng@tongji.edu.cn; 5 Research and Development Division, CYBERDYNE INC, Tsukuba 3050818, Japan; sato_naoto@cyberdyne.jp; 6 Research and Business Development Division, CYBERDYNE INC, 3013 Rotterdam, The Netherlands

**Keywords:** photoacoustic, LED, clinic, optical imaging

## Abstract

Photoacoustic imaging, with the capability to provide simultaneous structural, functional, and molecular information, is one of the fastest growing biomedical imaging modalities of recent times. As a hybrid modality, it not only provides greater penetration depth than the purely optical imaging techniques, but also provides optical contrast of molecular components in the living tissue. Conventionally, photoacoustic imaging systems utilize bulky and expensive class IV lasers, which is one of the key factors hindering the clinical translation of this promising modality. Use of LEDs which are portable and affordable offers a unique opportunity to accelerate the clinical translation of photoacoustics. In this paper, we first review the development history of LED as an illumination source in biomedical photoacoustic imaging. Key developments in this area, from point-source measurements to development of high-power LED arrays, are briefly discussed. Finally, we thoroughly review multiple phantom, ex-vivo, animal in-vivo, human in-vivo, and clinical pilot studies and demonstrate the unprecedented preclinical and clinical potential of LED-based photoacoustic imaging.

## 1. Introduction

Photoacoustic imaging (PAI) holds strong potential in providing structural, functional and molecular information on tissue, with scalable resolution and imaging depth [1]. Since the optical scattering is high in biological tissue, ballistic optical microscopic techniques cannot provide any useful information beyond a depth of 1 mm. PAI overcomes this difficulty since it involves acoustic detection and sound scattering in tissue is orders of magnitude lower than that of light. In PAI, short-pulsed light is irradiated on the tissue, and endogenous optical absorbers in the tissue absorb light resulting in a temperature rise [2]. This transient temperature rise results in thermoelastic expansion and produces light-induced ultrasound (US) waves, which then can be detected by US detectors placed on the skin surface for reconstructing an optical absorption map with acoustic resolution [3]. In a clinical context, PAI is easy to combine with US imaging and is capable of providing anatomical, functional, molecular, and metabolic information by utilizing the signature optical absorption contrast of the vasculature, hemodynamics, oxygen metabolism, biomarkers, and gene expression. Utilizing the useful information provided by PAI, a plethora of clinical applications have been explored in vascular biology [4,5,6,7], oncology [8,9], neurology [2,10,11], ophthalmology [12,13], dermatology [14,15], gastroenterology [16,17,18,19,20], osteology [21,22,23,24], and cardiology [25,26]. 

In laser-based PAI, where the tissue of interest is illuminated by a pulsed laser beam, the optical energy used usually ranges from tens to hundreds of mJ per pulse, with a typical pulse duration of 5–10 ns. Most of the commercial and research lab-made PAI systems utilize Q-switched Nd:YAG pumped OPO (optical parametric oscillator), Ti: Sapphire or dye laser systems. However, because of their high cost, larger footprint and strict requirement for eye-safety goggles and laser-safe rooms, these laser sources are not suitable for a clinical environment. Furthermore, the repetition rate of most high-power laser sources is relatively low (~10 Hz), which limits the imaging speed, especially when the signal-to-noise ratio (SNR) is not sufficient and frame averaging is a necessity. In recent years, laser diodes (LD) and light emitting diodes (LEDs) have been heavily explored to be used as an illumination source in PAI, resulting in portable, affordable and clinically translatable PAI systems [6,7,27,28,29,30,31,32,33,34,35,36,37]. LD offers a higher pulse repetition rate (PRR) (typical 2–4 KHz), average power around 6 W, and optical energy of around 0.56–2.5 mJ per pulse, but is only available at wavelengths greater than 750 nm [38,39]. Additionally, for an LD-based PAI system, laser-safe rooms and goggles are requirements, just as in the case of conventional laser sources. On the other hand, LEDs, which are available in a wide wavelength range (e.g., 470, 520, 620, 660, 690, 750, 820, 850, 940 and 980 nm) provide lower optical energy in the range of µJ per pulse, but at a higher repetition rate (~16 KHz) offering the possibility to average more frames without compromising on temporal resolution. Compared to fixed pulse widths in lasers, the optical pulse width of an LED/LD source can be tuned based on the required spatial resolution and imaging depth [39,40]. 

The pulse width of LED/LD sources is tens of nanoseconds, whereas that of solid-state lasers could be less than ten nanoseconds. The temporal pulse width imposes a limit to the spatial resolution of the imaging system. For example, the 35-ns pulse width of the 850-nm LED corresponds to a spatial resolution of 52.5 µm (=35 ns × 1500 µm/µs). The LD usually offers more energy than LEDs. An LD light source’s (Quantel, Bozeman, MT) pulse width, for example, can be tuned from 30 ns to 200 ns, with the pulse energy correspondingly changing from 1–4 mJ. As a limitation, the LED array can only reach up to 0.200 mJ per pulse (highest reported optical output for 850 nm LED arrays). LD, even though it can offer higher pulse energy, is the same as class-IV lasers in terms of optical coherence and subsequent eye/skin safety issues. Owing to its portability, affordability, imaging speed and safety aspects, LED-based PAI holds potential in real-time functional and structural characterization of tissue in various superficial and sub-surface imaging applications and also to accelerate the clinical translation of PAI. Typically used laser, LD and LED performance are listed in Table 1. 

In this paper, we review the progress of LED-based PAI technology and its potential preclinical and clinical applications.

## 2. Fundamental Development of LED-Based PAI Technology

PAI has already demonstrated its unparalleled potential in multiple preclinical and clinical applications and is quite mature in a research setting. At this point, this technology is facing an exciting transition from bench to bedside and LD- and LED-based systems are being explored heavily because of their portability, affordability, and ease of use in a clinical setting. LDs operating in pulsed mode have been investigated by different research groups for multiple point-of-care applications [36,37,38]. However, typical commercial pulsed LDs are available only in the near-infrared wavelength range, and combining multiple wavelengths in a handheld setting is a cumbersome process [41,42]. On the other hand, the LEDs could be fabricated to operate in a 400 nm to 1000 nm wavelength range (not continuously) with reasonable optical energy by developing arrays of multiple elements and overdriving them [43]. Within this wavelength range, PAI could provide high contrast for melanin, hemoglobin, and fat to an extent, making LEDs one of the ideal illumination sources for multispectral PAI of tissue up to a depth of 1–1.5 cm. 

An LED is a semiconductor device based on a p–n junction diode. A p-n junction diode is a two-terminal semiconductor device, which allows the electric current in only one direction and blocks the electric current in the opposite or reverse direction. If the diode is forward biased by applying a voltage, it allows the electric current flow. A small increase in voltage results in a significant change in current flow. Holes (from the P-type material) and electrons (from the N-type material) flowing across the junction promote strong electron–hole radiative recombination, resulting in the emission of a large number of photons [44]. Typically, LEDs are designed for continuous wave (CW) operation, but it is also feasible to drive them with pulsed current. In the pulsed mode, output energy of LEDs is dependent on the peak current, and this can be far higher than the CW rated current, especially if the duty cycle is kept low (<0.1%) to avoid any thermal damage. Since LEDs, when overdriven in a pulsed mode, can generate significantly higher optical output than in conventional CW operation, these types of overdriven pulsed-mode LEDs are often referred to as high-power LEDs. However, operating an LED at excessively high drive currents may lead to faster ageing due to heat generation, and this can even cause immediate failure of the LEDs. The quantum efficiency of the device will also drop with increasing current [45]. Considering this, it is important to design and develop efficient, safe electronic drivers and heat sinks to use LEDs in high-power mode.

About a decade ago, Hansen first proposed the use of LEDs working at a 627-nm wavelength as an inexpensive and compact excitation source for biomedical PAI [46]. In this proof-of-concept work, he demonstrated the feasibility of using LEDs as a light source in PAI for the first time. The basic idea of creating pulsed high-power LED is that, when the low-power LED is overdriven, they can deliver optical output that is far higher than their normal specifications in CW-operation [47,48,49,50]. The LED they used was Luxeon LXHL_PD09 which has been measured to yield approximately 250 mW of light output when supplied with 1 A DC current. A derivative of the MOSFET-based circuit presented by Alton and Raji was employed as a driver for generating pulsed current [51]. When the LED was supplied with 60-ns current pulses with peak value of 40 A, it was able to provide pulse energy of 400 nJ per pulse with pulse width of 60 ns, and light focusing was performed to generate the radiant energy required for generating a photoacoustic (PA) response. Also, 50,000 A-line signals were averaged to detect PA response from a non-realistic phantom. Based on the PRR of the proposed LED (200 Hz), this system requires 250 s to acquire an image, which was not good enough for imaging tissue in real-time. Owing to the advances in solid-state device technology and efforts of different research groups in the last decade, there were significant improvements in the performance of LEDs (improvement of pulse energy, PRR, etc.), which consequently resulted in the step-by-step development and commercialization of an LED-based PAI system that is comparable to a laser-based machine. 

In 2013, Allen et al. proposed the use of high-power LEDs (CBT−120 from Luminus) working at 400-nm to 65-nm wavelengths as an illumination source in biomedical PAI [43]. In this work, the pulse energy of the LED is increased to 22 µJ per pulse with a pulse width of 500 ns, by overdriving the LED elements by 10 times their rated current. The driver they used was made based on MOSFET, which is described in work of Chaney et al. [51]. As a result of increase in light energy, with the same PRR of 200 Hz, they were able to significantly reduce the frame averaging (by 1000 times), which is commendable. 

**Figure 1:**
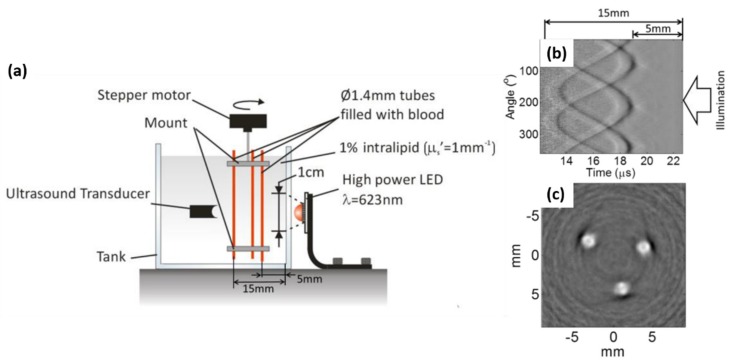
(**a**) PAI setup. (**b**) Time-resolved PA signals of three 1.4 mm tubes filled with human blood (35% haematocrit) and immersed in 1% Intralipid (μs’ = 1 mm^−1^). (**c**) Reconstructed PA image. Energy = 9 µJ, averaging image frames = 5000. Reprinted from Ref. [33].

In 2016, Allen et al. further improved their system performance by using high-power LED (SST−90 from Luminus) elements working at 400-nm to 650-nm wavelengths, driven by a commercial electronic driver (PCO−7120, Directed Energy, Inc., Loveland, CO, USA), which provided 9 µJ per pulse, with a pulse duration of 200 ns, a peak current of 50 A and a PRR of 500 Hz when overdriving LEDs by 20 times their nominal current [33]. They confirmed that the duty cycle was 0.01%, still below the 1% which has been previously reported as safe (no noticeable damage to the device) [45]. In this work, they first imaged a realistic tissue-mimicking phantom by averaging 5000 image frames and using a wide field illumination strategy. The best imaging depth they achieved was 15 mm in 1% intralipid (Figure 1).

The pioneering works mentioned above laid the foundation for LED-based PAI, especially demonstrating the feasibility of using visible light for PAI, which is not possible using lasers or LDs. Oxygen saturation imaging is one of the most important applications of PAI, and it is of paramount importance to have illumination wavelengths suitable for this. Considering the absorption peak for deoxy-hemoglobin (690 nm) and oxy-hemoglobin (850 nm), it was critical to develop LEDs in these wavelength ranges too. Considering this, there has been significant efforts from different researchers in this direction, which are detailed below. 

In 2016 and 2017, Agano et al. for the first time proposed the use of high-power LED arrays working at 850 nm with optical energy of 200 µJ per pulse and with a pulse duration of ~70–100 ns. They optimized the SNR by increasing the optical output, by developing highly noise-efficient front end electronics with multiple stages of amplification (~106 dB), and also by matching the US transducer’s frequency characteristics with LED light pulse width [34,35,52]. To increase the optical output, they developed LED arrays and further optimized them using hybrid techniques. The single LED element provided output energy of 0.024 µJ per pulse, with a pulse duration of 70 ns and 1 A DC-current. By developing LED elements with double stack structure, arranging them in an array, and applying 20 times the rated current, they were able to achieve 200 µJ per pulse at a wavelength of 850 nm. The repetition rate of this first commercial LED-based PAI system (AcousticX) was 4 KHz. In 2018, Zhu et al. improved the PRR of LEDs to 16 KHz and used the same system for demonstrating its dynamic structural and functional imaging capabilities [6]. The performance of the mentioned LED-based PAI technology is summarized in Table 2.

The AcousticX system was thoroughly characterized for its imaging depth, spatial resolution, frame rate, and oxygen saturation imaging accuracy in multiple studies. When using a 9-MHz US probe and 850-nm LED arrays, Xia et al. reported mean axial and lateral resolutions of 220 µm and 460 µm respectively, which was similar for both US and PA imaging. They also reported an imaging depth of 2.8 cm at an interleaved US and PA frame rate of 30 Hz. Imaging depth was further improved to 3.8 cm after averaging more frames, resulting in a final frame rate of 1.5 Hz. In another study, Hariri et al. reported mean axial and lateral resolution of 268 µm and 570 µm respectively, when using a combination of a 7-MHz US probe and 850-nm LED arrays. They also obtained an imaging depth of 3.2 cm (frame rate = 15 Hz) in their phantom study. In this molecular imaging study, they also measured the sensitivity of the system in detecting commonly used molecular contrast agents. The limits of detection for ICG, MB, and DiR were reported to be 9 μM, 0.75 mM, and 68 μM, respectively. Oxygen saturation imaging is one of the most important applications of PAI. Using a two-wavelength (750/850 nm) approach, Kalloor Joseph et al. evaluated the potential of AcousticX in oxygen saturation imaging using phantom and in-vivo small animal imaging experiments. Based on 28 human-blood-based in-vitro measurements, a standard error of 8.4% was observed between actual oxygen saturation values and the oxygen saturation image formed by the system. In the same work, they showed repeatability and reproducibility of oxygen saturation imaging using an in-vivo mouse oxygen breathing challenge experiment. 

From the beginning of 2017, there has been a tremendous push in this area and multiple studies using LED-based PAI were reported. In the next section, we will review the potential preclinical and clinical applications demonstrated using AcousticX, the commercially available LED-based PA and US imaging system. 

## 3. Preclinical and Clinical Applications of LED-Based PAI 

Several preclinical and clinical studies utilizing LED-based PAI are reviewed in this section to provide a perspective for future clinical translation.

### 3.1. Phantom and Ex-Vivo Studies 

#### 3.1.1. Guidance on Minimally Invasive Procedures with Peripheral Tissue Targets

**Figure 2:**
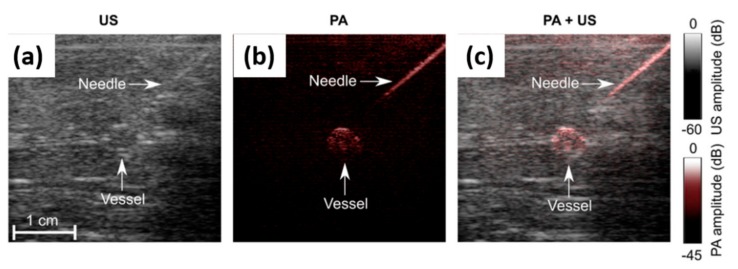
(**a**) US, (**b**) PA and (**c**) US-PA overlay images showing a medical needle inserted towards a vessel-mimicking phantom embedded in chicken tissue, respectively. Here, the uppermost 5 mm, which contained the US gel, is not shown. Reprinted from Ref. [32].

Precise and efficient device guidance is critical for minimally invasive vascular access procedures. US imaging is commonly used in clinics for this purpose, but the visualization of medical needles and tissue targets are often challenging [53]. PAI with high contrast for metallic needles and vasculature holds strong potential for guiding these needle-based vascular access procedures. However, the bulky and expensive solid-state laser that is required for tissue illumination is hindering the transition of PAI from bench to clinic, for this point-of-care application [54,55,56]. In 2018, Xia et al. used AcousticX and demonstrated the potential of LED-based PAI and US imaging in guiding minimally invasive procedures [32]. Their results demonstrated that LED-based PAI enabled needle visualization with SNRs that were 1.2 to 2.2 times higher than those obtained with US imaging, over insertion angles of 26 to 51 degrees. In the reported phantom study, an imaging depth close to 4 cm was achieved as shown in Figure 2a–c. In this work, they also demonstrated that LED-based PAI can visualize the superficial vasculature of the finger and wrist of a human volunteer in real time, along with conventional pulse-echo US. Based on these promising results, it can be concluded that LED-based PAI combined with US imaging holds potential in guiding minimally invasive procedures with peripheral tissue targets.

#### 3.1.2. Imaging of Human Placental Vasculature

**Figure 3:**
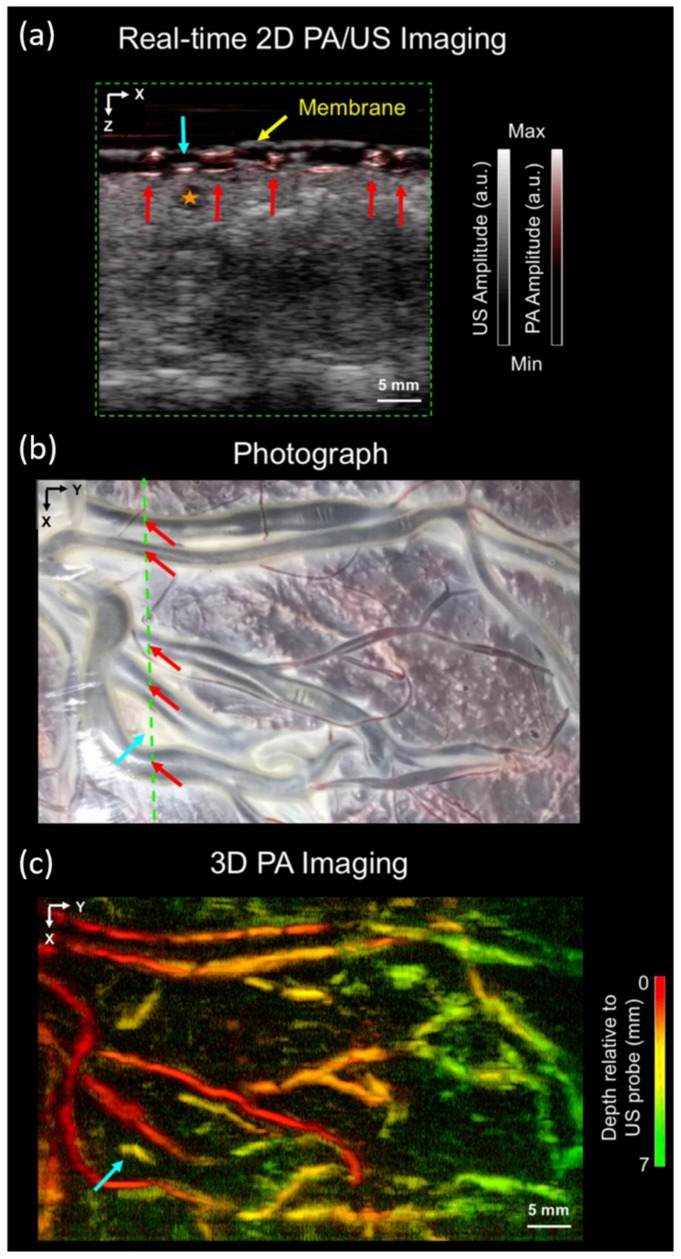
Wide-field PA-US images of the chorionic placental vasculature in an untreated part of a TTTS photocoagulated placenta. (**a**) 2D PA and US overlay image, acquired from a region corresponding to the green line in (**b**), which is a photograph of the imaged placenta. (**c**) 3D PA image displayed as a maximum intensity projection (MIP) of the reconstructed image volume. Reprinted from Ref. [64].

Accurate guidance is important not only for vascular access procedures, but also for minimally invasive fetal surgeries, for example, for the procedure performed to treat twin-to-twin transfusion syndrome (TTTS) [57]. TTTS is caused by imbalanced blood flow between twin vascular connections (anastomoses) in the placenta. If not treated in time, it will bring high morbidity and risk of death [58]. In current clinical practice of the TTTS procedure, in-vivo imaging of the placenta is performed using white-light fetal endoscopy and external B-mode US imaging. Both these methods do not provide sufficient contrast to visualize small anastomotic vessels below the surface of the chorionic placenta [59]. In 2018, Maneas et al. employed the LED-based PA imaging for wide field imaging of the human placental vasculature ex vivo, taking advantage of high optical absorption contrast offered by oxy- and deoxy-hemoglobin [60,61]. Previous studies have shown that PA signals from placental vessels can be detected using external clinical US probes [62,63]. Here, they used an LED-based PAI system (AcousticX) to image chorionic (fetal) superficial and subsurface vasculature in normal and TTTS-treated human placentas [64]. The LED-based PAI system based on a clinical US probe enabled fast interleaved 2D PA and US imaging (Figure 3). This work gives direct confirmation that volumetric LED-based PAI of the human placenta can generate detailed 3D maps of surface and subsurface vasculature up to a depth of around 7 mm. The authors also foresee that interventional PA imaging for visualizing the chorionic surface and superficial placental blood vessels with high spatial resolution may be valuable for minimally invasive fetal treatments.

#### 3.1.3. Imaging of Intraocular Tumors

**Figure 4:**
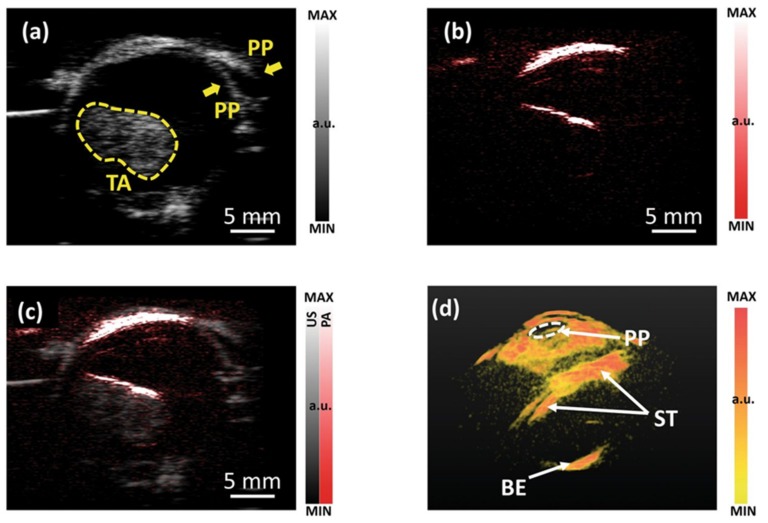
(**a**) 2D B-scan US image and (**b**) PA image of an ocular globe ex vivo with a tumor inside (confirmed in clinic). (**c**) PA and US combined image. (**d**) Perspective view of a 3D PA image of ocular globe with a choroidal melanoma tumor. PP-pupil. TA-tumor area. ST-the surface of the tumor. BE-the back of the eye. Reprinted from Ref. [6].

PA imaging has shown great potential in diagnosis and characterization of cancer, especially in applications like head and neck tumor imaging [65,66,67,68,69,70]. As a head and neck cancer, intraocular tumors are relatively rare, but life-threatening [70,71]. Xu et al. have demonstrated the ability of laser-based PA imaging to characterize intraocular tumors through molecular composition and structural heterogeneity [72]. Zhu et al. recently explored the feasibility of using LED-based PAI to image intraocular tumors in a complete human eyeball. Figure 4a,b shows 2D US and PA images of the eyeball of a choroidal melanoma tumor, respectively. US is shown in grayscale and PA is shown in pseudo-color. Figure 4c is the PA/US overlay image, offering complementary information. PA results show that LED-based PA imaging has sufficient penetration depth to cover the entire tumor volume. Figure 4d shows a 3D PA image of the eyeball. In this work, single-wavelength LED arrays were used to visualize vasculature. LED arrays with multiple wavelengths can be potentially used to observe individual molecular components in the future. These results reveal the potential of LED-based PAI in the broad area of cancer diagnosis and staging.

### 3.2. In-Vivo Preclinical Small Animal Imaging Studies

#### 3.2.1. Non-Invasive Monitoring of Angiogenesis

**Figure 5:**
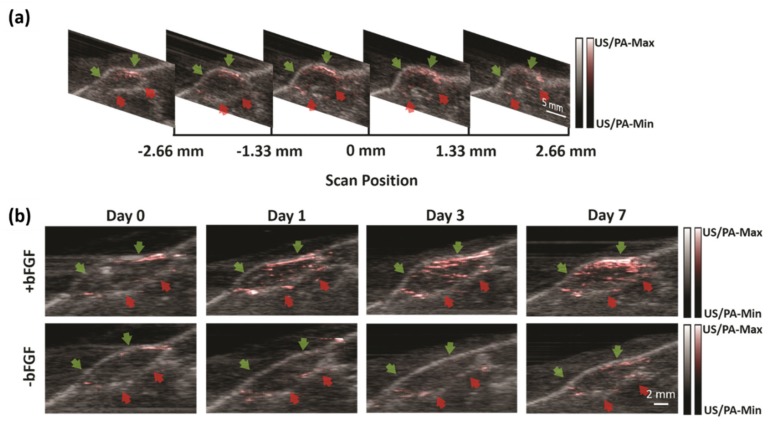
Longitudinal LED-based PA-US imaging of two subcutaneous implants. Green and red arrows indicate the upper and lower edges of the scaffold, as determined through the B-mode US. (**a**) A series of two-dimensional US-PA images from a +bFGF scaffold on day 7 at different scan positions. Note that only images within the range of −2.5 to 2.5 mm are used for MIP image. (**b**) A series of longitudinal MIP US-PA images of +bFGF and −bFGF scaffolds from the same mouse. PA intensity represented in red has the greatest difference on day 7. Reprinted from Ref. [7].

Vascularization of engineered constructs is required to integrate an implant within the host blood supply. The ability to non-invasively monitor neovascularization of an implanted construct is ultimately critical for translation. Laser speckle contrast analysis (LASCA), a widely used imaging technique within regenerative medicine, has high spatial resolution, but offers limited imaging depth and is only sensitive to perfused blood vessels [73,74,75]. In 2019, Zhu et al. used LED-based PA and US imaging to potentially solve this challenge in regenerative medicine [7]. They used an LED-based PA-US dual-mode system to image and monitor angiogenesis for 7 days in fibrin-based scaffolds, which were subcutaneously implanted in mice. Scaffolds, with or without basic fibroblast growth factor (bFGF), were imaged on days 0 (i.e., post implantation), 1, 3, and 7 with both LASCA and PA-US imaging systems (Figure 5). Quantified perfusion measured by LASCA and PA imaging was compared with histologically determined blood vessel density on day 7. Vessel density corroborated changes in perfusion measured by both LASCA and PA. PAI enabled delineation of differences in neovascularization in the upper and the lower regions of the scaffold. Overall, this study has demonstrated that PAI could be a noninvasive and highly sensitive method for monitoring deep-seated vascularization in regenerative applications.

#### 3.2.2. Noninvasive Imaging of Pressure Ulcers 

**Figure 6:**
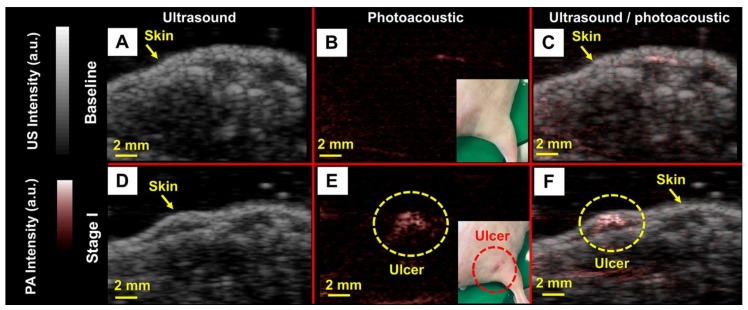
LED-based PA evaluation of pressure ulcers at stage I. (**A**) B-mode US image at baseline conditions when no pressure has been applied. (**B**) B-mode PA image at baseline at the same position as panel A. Minor PA signal is observed from the epidermis. The photographic inset shows the mouse in absence of ulcer. (**C**) B-mode PA/US overlay at baseline conditions. (**D**) B-mode US image at stage I. (**E**) B-mode PA image at stage I at the same position as panel D. We observed a 2.5-fold increase in PA intensity compared to baseline. The photographic inset shows the stage I ulcer. (**F**) B-mode PA/US overlay at stage I. The image depth is 1 cm and the scale bars are 2 mm. Reprinted with permission from Ref. [76].

Hariri et al. used PAI as a noninvasive method for detecting early tissue damage that cannot be visually observed. They used a mouse model of pressure ulcers by implanting subdermal magnets in the dorsal flank and periodically applying an external magnet to the healed implant site. The magnet-induced pressure was applied in cycles, and the extent of ulceration was dictated by the number of cycles. They evaluated these ulcers with LED-based PAI. Figure 6 shows baseline (top) and stage I ulcers (bottom) via US, PA, and US/PA overlay images obtained using LED excitation. The insets in Figure 6B–C are photographs of mice without and with pressure ulcers, respectively. They used a LED-based PAI system to detect early stage (stage I) pressure ulcers and observed a 2.5-fold increase in PA signal. Importantly, they confirmed the capacity of this technique to detect dysregulated skin even before stage I ulcers have erupted. They also observed significant changes in PA intensity during healing, suggesting that this approach can monitor therapy. These findings were confirmed with histology (not shown here). These results suggest that this PA-based approach might have clinical value for monitoring skin diseases, including pressure ulcers.

#### 3.2.3. Oxygen Saturation Imaging in Rheumatoid Arthritis Diagnosis

Multiwavelength LED-based PAI is a useful tool for functionally characterizing tissue in different clinical applications, for example, to obtain oxygen saturation. Hypoxia in the joints are biomarkers of Rheumatoid Arthritis (RA). The ability to accurately estimate the oxygen concentration makes multiwavelength PAI a potential tool for early detection of RA. Joseph et al. used in vivo animal study to find the capability of measuring the oxygen saturation using this system [77]. First, ex-vivo PA oxygen saturation imaging using human blood was validated against oximeter readings and further verified with in-vivo animal studies. The PA oxygen saturation estimation correlates with oximeter readings, which is confirmed with in-vivo studies. In the case of oxygen saturation, an approximately 5-mm imaging depth was achieved. This imaging depth is considered sufficient for RA imaging of the finger joint.

**Figure 7:**
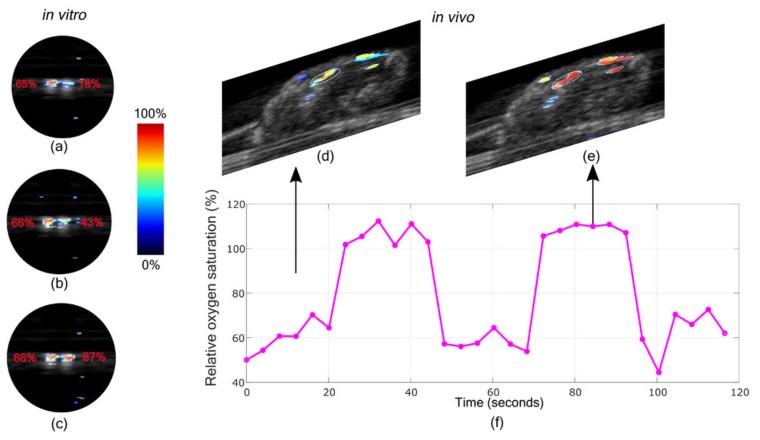
(**a**–**c**) The in-vitro study oxygen saturation map, with three different oxygen concentrations in the tube on the right, keeping oxygen unchanged in the left tube. The oxygen concentrations values in left and right tube: (**a**) 65% and 18%, (**b**) 66% and 43%, (**c**) 66% and 87%, (**d**) PA and (**e**) US imaging of mouse knee. In-vivo oxygen saturation images of mouse thigh muscle during high and low cycles of oxygen concentration. (**f**) Average oxygen saturation in the region of interested plotted over time. Reprinted from Ref. [77].

Results from the blood oxygen saturation imaging are shown in Figure 7. Figure 7a–c shows an oxygen saturation map of two tubes, one with normal blood (oxygen concentration approximately 65%) and another with blood having 18%, 43% and 87% oxygen concentration respectively. This correlates with the estimated oxygen saturation generated by the imaging system. Further, in-vivo imaging of the thigh muscle of the mouse is shown in Figure 7d–f. In the case of the mouse breathing normal air, an average oxygen concentration of 61.1% was observed and with 100% oxygen it was 105.3% using the system. This shows that the system can provide a fairly accurate estimate of oxygen saturation with in-vivo imaging. Through their results, they have shown that early stage rheumatoid arthritis changes such as synovial angiogenesis and hypoxia can be imaged using the LED-based PA imaging system. The results give a direct confirmation that multispectral LED-based PA holds potential in early detection and staging of RA in animal studies.

#### 3.2.4. Molecular Imaging: Detection and Monitoring of Reactive Oxygen and Nitrogen Species

Reactive oxygen and nitrogen substances (RONS) regulate important functions in living systems. Endogenous RONS contribute to signal transduction, smooth muscle relaxation, and blood pressure regulation [66,67]. RONS disorders can cause diseases, such as cancer, and RONS detection can be used to diagnose and treat infections and various diseases [68,69,70].

**Figure 8:**
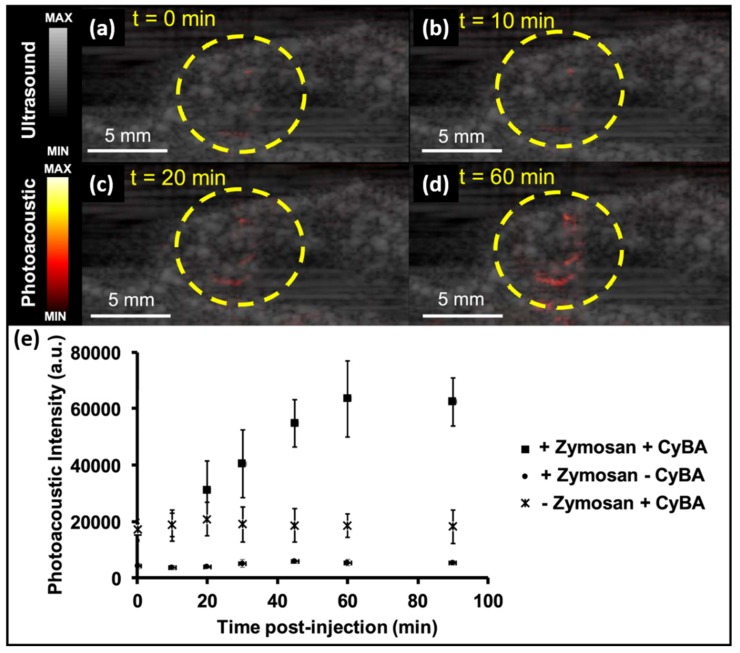
In-vivo PA evaluation of CyBA. US-PA image at (**a**) baseline, (**b**) 10, (**c**) 20, and (**d**) 60 min after CyBA injection. (**e**) Quantitative analysis of PA intensity as a function of time post-injection of CyBA. Reprinted from Ref. [29].

In 2019, Hariri et al. reported on molecular imaging of RONS using near-infrared absorbing small molecules (CyBA) and an LED-based PA imaging system [29]. They evaluated CyBA’s ability to measure inflammation in mice. Figure 8a—d shows the US/PA images from the injection location of CyBA and zymosan at 0, 10, 20, and 60 min, respectively (Figure 8). These figures show an increasing trend in PA signals due to the diffused probe (CyBA) at the region of interest (ROI), marked using a yellow circle. Figure 8e represents the quantitative analysis of CyBA’s in-vivo PA imaging assessment. These results indicated a gradual increase in PA intensity, and a ~3.2-fold increase was quantified 90 min after CyBA injection. It also shows the flat PA intensity trend of separated zymosan and the dye.

As we know, the LED output energy is about 1000-fold lower than that of conventional Nd:YAG lasers. However, it turns out to be a positive factor here: the higher power generated by Nd:YAG lasers bleach dyes, but not the light from LEDs. Therefore, their work not only revealed the sensitivity of PA in detecting RONS, but also emphasized the practicality of LED-based PAI in the clinic for molecular imaging applications involving dyes. In this work, they demonstrated that advantages of LED as an illumination source may help to accelerate the clinical translation of PAI. It is foreseen that LED-based PAI and reported probes can be used for clinical monitoring of RONS, especially for keloid diagnosis and for drug toxicity studies [78].

#### 3.2.5. Imaging of Tumor Vasculature Using Contrast Enhancement

Contrast enhancement is prevalently used in vasculature imaging. Xavierselvan et al. tested the LED-based PAI system for its ability to image the vasculature in the tumor using contrast enhancement [79]. For their study, they used subcutaneous head and neck tumor (FaDu) xenografts in nude mice. When the tumor size reached about 100 mm^3^, the tumors were imaged using the AcousticX system. The image shows heterogenous vascular density in the tumor which demonstrates the ability of the LED-based PAI system to obtain vascular information from tissues that are more than 1 cm deep. 

**Figure 9:**
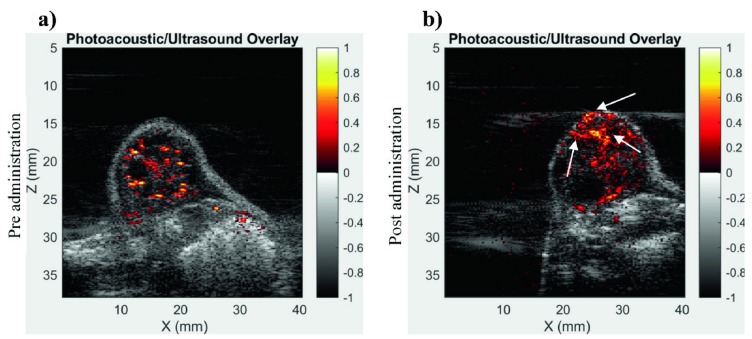
Contrast enhancement in LED-based PAI using exogeneous contrast agents. Combined overlay of PA and US image of subcutaneous FaDu tumor in mice before (**a**) and after (**b**) the NC dye administration. PA images were acquired using 850 nm LED light source (areas with greater contrast is shown with white arrows). Reprinted with permission from Ref. [79].

Through their study, they found LED-based PAI was a more affordable option for various research groups to avail the opportunity of utilizing the technology to understand nanoparticle or drug uptake non-invasively at high resolution. Figure 9 demonstrates the capability of the LED-based PAI system in imaging a naphthalocyanine (NC) dye, which has strong absorption in the NIR region (~absorption peak at 860 nm). Before and after 100 µL of NC dye was injected intratumorally into the FaDu tumor interstitium, the distribution of dye inside the tumor was imaged respectively. The NC dye has distributed into almost all parts of the tumor, but a strong PA signal was received from the top of tumor, which is potentially due to these areas receiving stronger light energy. 

#### 3.2.6. Imaging of Molecular-Labelled Cells

Hariri et al. have previously used PA imaging for stem cell imaging [80,81]. In a recent work [27], they used labeled cells to understand the in-vivo molecular imaging performance of AcousticX. They used DiR, which is an effective contrast agent for cellular imaging. The cells used here were human mesenchymal stem cells (HMSCs; Lonza, PT−2501, NJ, USA).

**Figure 10:**
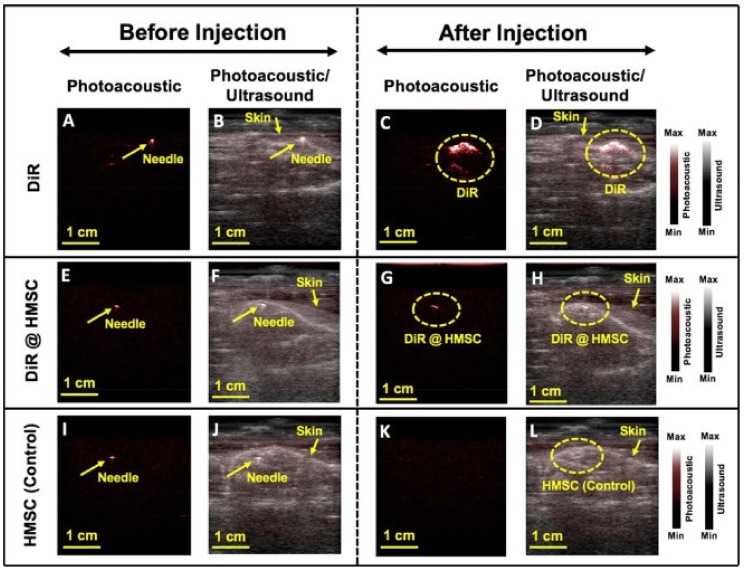
Images were separated in three groups: injecting DiR (**A**–**D**), DiR + HMSC (**E**–**H**), and HMSC (**I**–**L**). Each group contains PA and overlaid PA-US images before and after injection. Needles marked in images are subcutaneously injected on spinal cord area before DiR injection. The needle has a strong PA signal. Reprinted from Ref. [27].

Figure 10 shows PA images before and after injection of DiR, DiR + HMSC, and HMSC, respectively. The needle generates a strong PA signal and simultaneous US acquisition, offering detailed structural information along with the functional details from DiR-labeled cells. A strong PA signal in the presence of DiR is visible in Figure 10D. A signal increase is also visible when HMSC is labelled using DiR, as shown in Figure 10H. Unlabeled HMSCs were also injected as control but there was no increase in PA signal. This study showed the potential of LED-based PA in cellular imaging.

### 3.3. In-Vivo Human Volunteer Studies

#### 3.3.1. Imaging of Peripheral Microvasculature and Function

**Figure 11:**
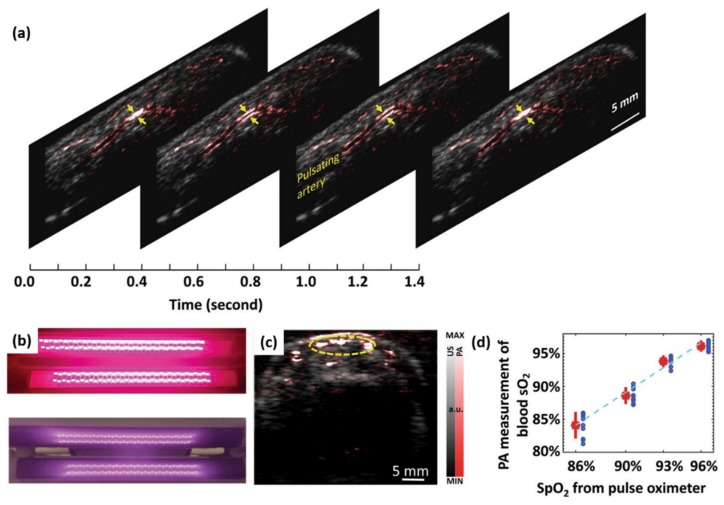
(**a**) Four frames from a PA imaging video (index finger, longitudinal section) presenting the pulsation of an artery marked by the arrows. (**b**) Photo of a pair of dual-wavelength LED bars that emits 690-nm and 850-nm light alternatively. (**c**) PA-US combined image showing a cross-section of the vasculature in a human finger. (**d**) Correlation between the LED-PAI-based blood sO2 in the finger and the SpO2 readouts from a pulse oximeter. Reprinted from Ref. [6].

Hypoxia is an important biomarker that reflects the occurrence and development of many diseases, such as cancer [82]. Multispectral PA imaging has been demonstrated to detect the relative hemoglobin oxygen saturation and hypoxia in biological samples in vivo in a non-invasive manner by detecting the spectral differences between oxygenated hemoglobin and deoxyhemoglobin [83,84,85]. Figure 11a shows LED-based PA and US B-scan images of a human finger along the sagittal section, which is from our recent work [6]. To show the arterial pulsation (shown by the arrow), four frames of the movie are shown. In this work, we also explored the feasibility of LED-based PA imaging to measure blood oxygen content on human finger blood vessels using a pair of two-wavelength LED bars (850 nm and 690 nm), as shown in Figure 11b [6].

Functional PA imaging of blood oxygenation saturation in blood vessels was performed on the index fingers of volunteers. Figure 11c is an example of a PA 2D image resolving blood vessels in an axial view of a finger, which is overlaid on a US image in grayscale. In order to quantify the functional information of PA imaging results, the oxygenation saturation levels of pixels in the ROI are averaged, as shown by the yellow dotted circle. The quantitative PA measurement of blood oxygenation saturation in the finger is then correlated with the reading of a pulse oximeter, which is the gold standard. As shown in Figure 11d, the blood oxygenation measured by LED-PAI and the reading of the pulse oximeter have a good correlation (R-squared ~ 0.98).

#### 3.3.2. Simultaneous Imaging of Veins and Lymphatic Vessels

**Figure 12:**
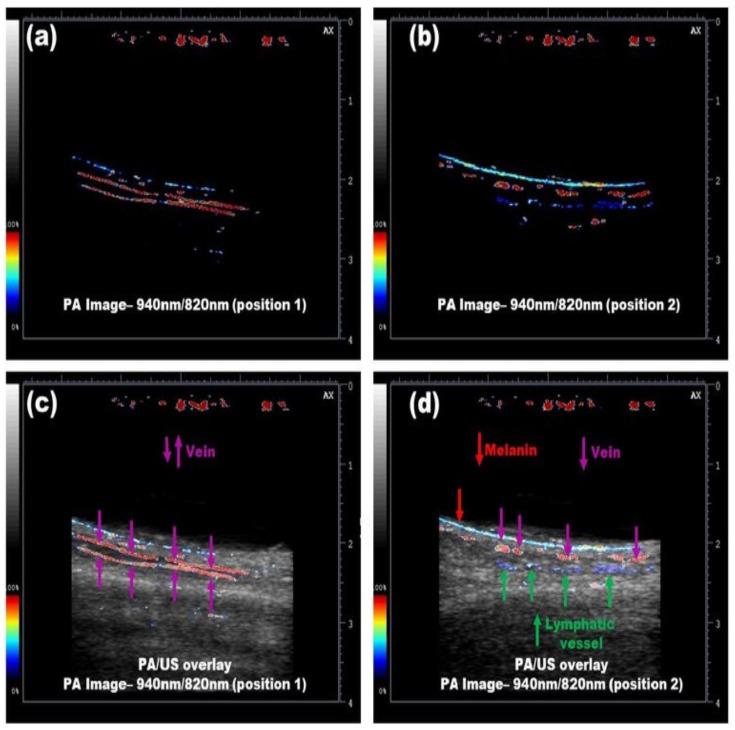
Real-time imaging of lymphatic vessels and veins simultaneously using LED-based PAI. (**a**) PA image: 940 nm/820 nm image generated when the probe was aligned to a vein (position 1); (**b**) PA image: 940 nm/820 nm image generated when the probe was aligned to a lymphatic vessel (position 2); (**c**) 940 nm/820 nm PA image overlaid on US image when the probe was in position 1; and (**d**) 940 nm/820 nm PA image overlaid on US image when the probe was aligned to position 2. Reprinted from Ref. [86].

Using dual-wavelength LED arrays working at 820/940-nm wavelengths, Kuniyil Ajith Singh et al. demonstrated that LED-based PAI could differentiate veins and lymphatic vessels (after injecting ICG) in human volunteers [86]. Utilizing a simple image division algorithm (assuming that ICG will not absorb 940-nm light), they showed that LED-based PAI can clearly differentiate veins and lymphatic vessels in real time, as shown in Figure 12. This multispectral PA/US approach holds strong potential in guiding procedures like lymphaticovenous anastomosis, where it is crucial to differentiate venous blood and lymphatic vessels.

#### 3.3.3. Full View Tomography of Finger Joints

**Figure 13:**
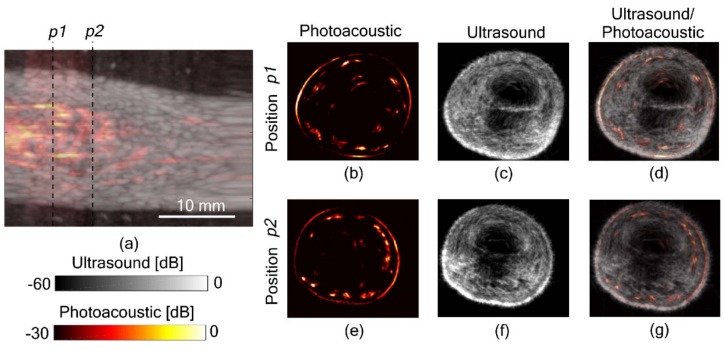
In-vivo finger joint tomographic imaging using multi-angle spatial compounding. (**a**) Overlaid LED-based PA and US MIP image showing finger joint from a linear scan. (**b**) PA, (**c**) US and (**d**) combined tomographic images of the finger joint (p1). (**e**) PA, (**f**) US and (**g**) combined tomographic images 5 mm in front of the joint (p2), respectively. Reprinted from [87].

It is well known that 2D PA and US imaging using linear arrays suffers from limited-view artifacts. Additionally, due to the directivity limitations of US transducers, there is a high probability of loss of information. One way to overcome this limitation is to scan around the object of interest and generate full-view 3D tomographic images with higher spatial resolution and no limited view problems. However, most of the commercially available and lab-made 3D PA/US tomography systems utilize pulsed lasers, which are expensive, bulky and not suitable for a point-of-care setting. Joseph et al. recently demonstrated the possibility to generate full view LED-based 3D PA and US tomographic images of human finger joints (Figure 13) [87,88]. This proof-of-concept study gives a direct confirmation that inexpensive and portable LEDs along with commercially available linear array US probes can generate high-quality 3D tomographic images with rich information suitable for multiple point-of-care clinical applications. In another promising study, Agrawal et al. developed a multispectral molecular LED-based 3D PA tomography system and demonstrated the potential in unmixing and separating three optical absorbers (melanin, methylene blue and ICG) embedded inside a tissue mimicking phantom [89].

### 3.4. Clinical Pilot Studies

#### 3.4.1. Imaging of Inflammatory Arthritis

Since PAI can detect hyper-vascularization and hypoxia, two key early markers of rheumatoid arthritis, it has been explored extensively in multiple preclinical and early clinical pilot studies [90,91,92,93,94]. Relatively small joints are usually first affected by inflammatory arthritis, and PAI in combination with conventional US imaging holds strong potential in this area. Early results from animal models and human subjects clearly indicate that PAI is expected to be translated soon to clinics as an affordable point-of-care tool for early detection of inflammatory diseases [95,96,97,98,99]. Janggun et al. explored the feasibility of using an LED-based PA system to detect inflammation of soft tissues surrounding joints, and the ability to distinguish arthritic joints from normal ones by assessing enhanced blood signal in synovial tissue [31].

**Figure 14:**
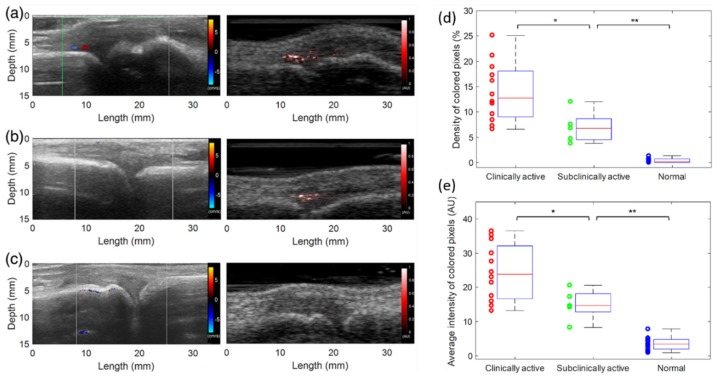
US Doppler (left) and PA (right) images acquired from LED-based system from three groups: (**a**) clinically active arthritis joints, (**b**) subclinical active arthritis joints, and (**c**) normal healthy joints). (**d**) The quantified results showing the density of colored pixels and (**e**) the average intensity of colored pixels in PA images of the three groups. * *p* < 0.05, ** *p* < 0.005. Reprinted from Ref. [31].

Figure 14 shows representative US Doppler and PA images of the three groups compared in this study, including clinically active arthritis joints, subclinical active arthritis joints, and normal healthy joints. Figure 14a shows a case of a clinically active case of arthritis, with hyperemia seen at the same location in both Doppler and PA images. Figure 14b shows a case of subclinical active arthritis, in which hyperemia is only visible in the PA image of the patient’s metacarpophalangeal (MCP) joint, but not in the US Doppler image. The normal subjects also underwent US Doppler scans as the patient volunteers, followed by scanning of same location of joints using LED-based PA imaging. When compared with the results of arthritic joints, no significant hyperemia was found in the synovium area of normal subjects, which has also been confirmed by US Doppler imaging. Figure 14c shows the normal situation; no hyperemia was seen in the US Doppler or PA image. Finally, Figure 14d,e shows the statistical results indicating there is significant difference between different groups, confirmed using a two-tailed *p*-test. This clinical pilot study gives a direct confirmation that LED-based PAI offers higher sensitivity to angiogenic microvasculature than US doppler imaging, the current gold standard.

#### 3.4.2. Diagnosis and Treatment Monitoring of Port Wine Stain

Port wine stain (PWS) is a benign capillary vascular malformation, with significant social and emotional impact. In a recent clinical pilot study, Cheng et al. demonstrated that LED-based PAI can be used as a point-of-care tool for clinical evaluation and treatment monitoring of PWS disease [100]. We foresee that LED-PAI will a have profound impact in this application, in which vascular contrast with depth information (~1 cm) is key for good diagnosis.

**Figure 15:**
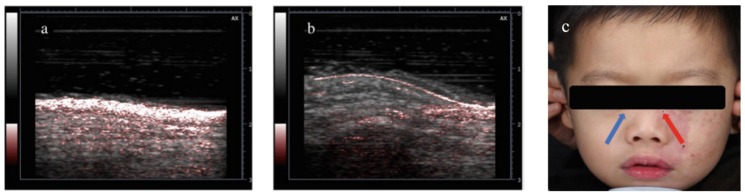
Typical PA/US overlay image of (**a**) PWS region (ROI) and (**b**) control region (HR). (**c**) Photograph of a PWS patient with imaging assisting marks. Reprinted with permission from Ref. [101].

PWS is categorized as a benign capillary vascular malformation, which is difficult to cure. In general, PWS appears on the face, but it can affect other areas of the body too. The affected skin surface may thicken slightly and develop an irregular, pebbled surface in adulthood. PWS’s cosmetic appearance causes substantial mental stress for the patients. In the study by Cheng et al., 22 patients were enrolled and separated into two groups based on their age (group 1: 3–6 years, group 2: above 6 years). The representative PA/US images (PWS region and normal region with clear difference in vascular contrast) are shown in Figure 15, along with the masked photograph of patient. The significant difference between the two different age groups also corresponds well with the given knowledge of PWS disease. Based on this result, it is clear that PAI as an imaging tool holds good potential in evaluation of PWS. Through this clinical pilot study, they demonstrated for the first time that LED-based PAI can be used as a point-of-care tool for clinical evaluation and PDT-treatment monitoring of PWS disease.

## 4. Discussion

In this paper, we first reviewed the historical development of LED-based PAI, starting from the first report on single-point measurements to latest clinical pilot studies using high-power LED arrays. In the span of 10 years, there has been significant growth in this field, especially with the improvement of pulse energy (nJ to hundreds of μJ) and PRR (200 Hz to 16,000 Hz) of LEDs and also the advancements in low-noise data acquisition electronics. All these developments have resulted in commercialization of the technology, and it is worth mentioning that LED-PAI is now capable of functional imaging (oxygenation and blood flow imaging) of superficial and sub-surface tissue (more than 1 cm) at frame rates unachievable for laser-based systems (500 Hz). Even though LED-based PAI cannot be used for applications requiring larger imaging depth (for example, a full breast), it holds potential in several superficial imaging applications, specifically in rheumatology and dermatology. 

Compared to solid-state lasers, the energy level of LED is two orders lower. However, the PRR of LED is much higher than that of a laser, which then can largely benefit the imaging quality by averaging more frames. Besides the emission energy level, another major difference between the pulsed LEDs and the solid-state lasers is the temporal pulse width. The pulse width of LED is tens of nanoseconds, whereas that of the solid-state lasers could be less than ten nanoseconds. The temporal pulse width imposes a limit on the spatial resolution of the imaging system. For example, the 70-ns pulse width of the 850-nm LED corresponds to a spatial resolution of 105 µm (=70 ns × 1500 µm/µs). This point, however, turns out to benefit the detection efficiency, especially when using bandlimited US probes for detection. The PA signal generated by a solid-state laser with a pulse width of 3.5 ns has a frequency component up to almost 300 MHz, in which anything above ~12 MHz will not be detected using a conventional mid-frequency range US probe. For a typical 5-MHz commercial US probe with 80% detection bandwidth, when using a 3.5-ns laser pulse, PA signal detection efficiency is 40 times less than the attainable efficiency when using an LED array generating 100-ns light pulses [102]. The resolution offered by a typical 5–10-MHz clinical US probe is 200 µm. Therefore, the pulse width of the LEDs can potentially be extended to 100 ns without affecting the spatial resolution (70 ns setting is used in all the studies reported in this paper).

In the second part of this paper, we reviewed some of the preclinical and clinical applications reported using LED-based PAI (in sequence of phantom, ex-vivo, small animal, and human in-vivo studies) as listed in Table 3. It is clear from the results that multispectral LED-based PA and US imaging holds strong potential in multiple applications, for example, guiding minimally invasive procedures, blood oxygen saturation imaging, diagnosis and staging of inflammatory arthritis, peripheral vascular assessment, guidance of surgical procedures like lymphaticovenous anastomosis, etc. In all the studies, an imaging depth of 0.5–1 cm was achieved at 10-Hz US and PA frame rates, which is good enough for multiple clinical applications. However, it is of paramount importance to improve the imaging depth for exploring more clinical applications, especially in the area of breast imaging and cardio-vascular medicine. To solve this issue and accelerate the clinical translation of LED-based PAI, several reconstruction and image processing techniques have been reported recently [103,104,105]. Use of clinically approved contrast agents (for example, ICG) may also be also useful to enhance the imaging depth. 

The LED-based PA imaging system described here (AcousticX) is safe for both skin and eye exposure. Since LED emissions are incoherent, the ANSI safety limits for collimated laser beams do not apply. Instead, the international electrotechnical commission (IEC) 62471 is followed. According to IEC 62471, the exposure limit for skin is based on thermal injury due to the temperature rise in tissue. Assuming that the illumination on the same skin area lasts continuously for 5 s using two 850-nm LED bars working at a 4-KHz pulse repetition rate, the estimated exposure is 4.57 × 10^3^ W·m^−2^, which is below the thermal hazard limit for skin of 5.98 × 10^3^ W·m^−2^. For eye safety, two aspects need to be considered, which are retinal thermal hazard exposure limit (weak visual stimulus) and infrared radiation eye safety limit. Assuming a continuous illumination at the front of the eye for 5 s using two 850-nm LED bars working at a 4-KHz pulse repetition rate, the estimated exposures are 2.92 × 10^3^ W⋅m^−2^⋅sr^−1^ for retinal thermal exposure and 4.57 × 10^3^ W⋅m^−2^ for infrared radiation exposure, both lower than the safety limits for eye of 1.34 × 10^5^ W⋅m^−2^⋅sr^−1^ and 5.38 × 10^3^ W⋅m^−2^, respectively.

To date, the maximum imaging depth achieved by LED-based PA imaging in an in-vivo setting is 1 cm. Even though this is encouraging considering the low pulse energy, it is important to improve LED optical output for better usefulness in more clinical applications. To an extent, signal averaging helps to improve SNR without effecting frame rate. However, if the magnitudes of the acoustic signals generated by the weak illumination are significantly below the noise-equivalent pressure level of the US probe, averaging will not effectively improve SNR. In terms of translational potential, addition of PA imaging to a clinical US system will have relatively easier clinical acceptance. Considering the acoustic bandwidth limitation of conventional pulse-echo US probes, we believe that the lower pulse width (70 ns is the setting used in all the applications reported in this paper) of LEDs is not a bottleneck. We foresee work on two aspects to improve the imaging depth: (1) use of nanostack technology to squeeze more light out of LED elements, with multiple p-n junctions embedded in the epitaxial layer, increasing the region’s light generation and thereby leading to a higher optical output, and (2) improving the driving electronics to increase the PRR further, consequently resulting in the possibility to average more frames and improve SNR without losing temporal resolution. LED might compete with LD with market share during clinical translation, but will not have much overlap with Nd: YAG OPO-based PAI system which focus on deep-tissue applications. However, LED-PAI may be a suitable option for point-of-care applications like guidance of peripheral vascular access procedures, rheumatoid arthritis screening, PWS diagnosis and treatment monitoring, etc. 

Looking into the future, it is foreseen that advances in high-power LED technology, mainly driven by the lighting industry, and significant developments in machine/deep learning and signal processing algorithms will increase the use of LEDs in the context of PAI. LED arrays in different shapes could be also developed to find different applications, for example, developing ring-shape LEDs for breast 3D imaging and coupling light into optical fibers for minimally invasive and endoscopic procedures. Worth mentioning here, if sufficient focusing can be achieved, LEDs will be the ideal candidate for acoustic-resolution PA microscopy (PAM). There have also been some recent reports in this direction. In 2017, Dai et al. presented a PAM system based on miniature LEDs working at a 405-nm source, which showed the capability of in-vivo mapping of vasculature networks in biological tissue [106,107]. They used a high-power LED (power~1.2 W) working at 405 nm wavelength, and a pulse width of 200 ns. The repetition rate was extended to 40 KHz towards meeting the high demand of scanning speed in a PAM setting. They acquired a complete PAM image in vivo in about 1 h, which is an encouraging result for the first LED-based PAM proof-of-concept study, but this temporal resolution is not good enough for clinical studies. On the other hand, for optical resolution PAM, the use of LEDs is likely to be challenging, as it would be difficult to achieve the necessary micron-scale diffraction-limited spot sizes. It would be also interesting to develop new low-frequency US probes (2–3 MHz) with ultra-high bandwidth and sensitivity to achieve higher imaging depth without compromising spatial resolution. 

## 5. Conclusions

The use of LEDs as an illumination source introduces some limitations. First, the LEDs cannot be spectrally tuned, which eliminates the possibility of PA spectroscopic applications in which multiple chromophores are involved. Second, the pulse width of the LED is low when compared to a laser (30–100 ns), which affects the stress confinement satisfaction and can impact the efficiency of acoustic wave generation. Third, LEDs have low optical output power—this can limit penetration depth at higher frame rates. However, LED-based PAI systems offers several advantages, including a significant reduction in cost, smaller footprint, no requirement of laser calibration and monitoring, and no need for optical goggles or light-tight shields. Thus, LED-based systems are not only suitable for point-of-care non-invasive applications complementing US imaging, but also ideal for personalized or wearable PA equipment, and we foresee that this technology could additionally have broad utility in a number of therapeutic drug monitoring applications. 

With wide optical wavelength range, flexible pulse-width setting, small footprint, low cost, and energy efficiency, LED-based PAI holds strong potential in functional and molecular preclinical and clinical imaging. We foresee that the addition of LED-based PAI to conventional US imaging in a clinical scanner will have a huge impact in point-of-care diagnostic imaging and also accelerate the clinical translation of PAI. 

## Figures and Tables

**Table 1:** Comparison of pulsed laser, laser diodes and LED [6,7,27,28,29,30,31,32,33,34,35,36,37,38,39,40].

	Energy (mJ)	PRR (Hz)	Pulse Width (ns)	Cost *	Advantages	Disadvantages
Laser	5 ~120	~10	<10	$70–200 K	Powerful, ~5 cm penetration depth, tunable wavelength	Bulky size, eye protection and laser safe rooms needed
LD	0.5–2.5	~1 K−6 K	30–200	~$10–25 K	Integration in a handheld probe feasible, high PRR	Limited penetration depth, eye protection and laser safe rooms needed, wavelength tuning not possible
LED	0.2	~200–16 K	30–100	$10–15 K	Integration in a handheld probe feasible, high PRR, no need for laser-safe rooms or eye-safety goggles	Limited penetration depth, wavelength tuning not possible

* Cost includes the driving electronics and may vary based on different features, number of wavelengths etc. Integration to a US probe may also involve extra development cost.

**Table 2:** Summary of the Fundamental development of LED-based PAI technology.

Year	Authors	Pulse Width (ns)	Peak Current (A)	Pulse Energy (mJ)	Repetition Rate (Hz)	Wavelength (nm)
2011	Hansen et al. [46]	60	40	0.0004	200	627
2013	Allen et al. [43]	500	200	0.0022	200	623
2016	Allen et al. [33]	200	50	0.0009	500	623
2016–2017	Agano et al. [34,35,52]	70 *	15–20	0.15–0.2 **	4000	850
2018	Zhu et al. [6]	70 *	20	0.2 **	4000–16,000	850

* Pulse width is tunable from 35–150 ns and above measurements are with 70 ns; ** value is measured from the arrays of the LED.

**Table 3:** Summary of preclinical and clinical applications of LED-based PAI.

Target	Application		Depth (mm)	Contrast Agent	Wavelength (nm)
Medical needles, Vasculature	Guidance of minimally invasive procedures with peripheral tissue targets [32]	Phantom and ex vivo studies	38	N/A	850
Vasculature	Imaging of human placental vasculature [64]		7	N/A	850
Tumor	Imaging of intraocular tumors [6]		10	N/A	850
Vasculature	Non-invasive monitoring of angiogenesis [7]	Animal in vivo	10	N/A	850
Ulcer	Noninvasive imaging of pressure ulcers [76]		10	N/A	690
Oxygen saturation	Oxygen saturation imaging in Rheumatoid arthritis [77]		5	N/A	750/850
Molecular	Detection and monitoring of reactive oxygen and nitrogen species [29]		10	CyBA	850
Tumor/Contrast agents	Imaging of tumor using contrast enhancement [79]		10	NC	850
Cells/ Contrast agents	Imaging of molecular-labelled cells [27]		10	DiR	850
Vasculature	Imaging of peripheral microvasculature and function [6]	Healthy human in vivo	10	N/A	690/850
Vasculature	Simultaneous imaging of veins and lymphatic vessels [86]		10	ICG	940/820
Finger joints	Full view tomography of finger joints [87]		5	N/A	850
Finger joints	Imaging of inflammatory arthritis [31]	Patient in vivo	5	N/A	850
Skin	Imaging of port wine stain [101]		10	N/A	850
